# Functional characterization of a *CDKN1B* mutation in a Sardinian kindred with multiple endocrine neoplasia type 4

**DOI:** 10.1530/EC-14-0116

**Published:** 2014-12-17

**Authors:** Elena Pardi, Stefano Mariotti, Natalia S Pellegata, Katiuscia Benfini, Simona Borsari, Federica Saponaro, Liborio Torregrossa, Antonello Cappai, Chiara Satta, Marco Mastinu, Claudio Marcocci, Filomena Cetani

**Affiliations:** 1 Endocrine Unit 2, Department of Clinical and Experimental Medicine, University Hospital of Pisa, University of Pisa, Via Paradisa 2, Pisa, Italy; 2 Endocrinology Unit, Department of Medical Sciences ‘M Aresu’, University of Cagliari, Cagliari, Italy; 3 Institute of Pathology, Helmholtz Zentrum Muenchen, Neuherberg, Germany; 4 Department of Surgical Medical and Molecular Pathology and Critical Area, University of Pisa, Pisa, Italy

**Keywords:** primary hyperparathyroidism, parathyroid tumorigenesis, MEN1, P27

## Abstract

Inactivating germline mutations of the *CDKN1B* gene encoding the nuclear cyclin-dependent kinase inhibitor P27^kip1^ protein have been reported in patients with multiple endocrine neoplasia type 4 (MEN4), a MEN1-like phenotype without *MEN1* mutations. The aim of this study was to characterize *in vitro* the germline *CDKN1B *mutation c.374_375delCT (S125X) we detected in a patient with MEN4. The proband was affected by primary hyperparathyroidism due to multiglandular parathyroid involvement and gastro–entero–pancreatic tumors. We carried out subcellular localization experiments by transfection with plasmid vectors expressing the WT or mutant* CDKN1B* cDNA into the eukaryotic human cervix adenocarcinoma (HeLa) and GH3 cell lines. Results from western blotting studies indicated that fusion proteins were expressed at equal levels. The mutated protein was shorter compared with the WT protein and lacked the highly conserved C-terminal domain, which includes the bipartite nuclear localization signal at amino acids 152/153 and 166/168. In HeLa and GH3 cells, WT P27 localized in the nucleus, whereas the P27_S125X protein was retained in the cytoplasm, predicting the loss of tumor-suppressive function. The proband's tumoral parathyroid tissue did not show allelic loss, because both WT and mutant alleles were determined to be present by sequencing the somatic DNA. Immunohistochemistry revealed a complete loss of nuclear expression of P27 in a parathyroid adenoma, which had been removed by the second surgery in the patient. In conclusion, our results confirm the pathogenic role of the c.374_375delCT* CDKN1B *germline mutation in a patient with MEN4.

## Introduction

Multiple endocrine neoplasia type 1 (MEN1, OMIM #131100) is a rare autosomal dominant endocrine disorder characterized by the occurrence of parathyroid adenoma/hyperplasia, duodeno–pancreatic neuroendocrine tumors (NETs), and anterior pituitary tumors in the same individual (1). A minority of affected patients may also develop a wide spectrum of endocrine and non-endocrine manifestations, such as adrenal cortical tumors, foregut carcinoid tumors, angiofibromas, collagenomas, and lipomas, thus contributing to the heterogeneity of the phenotypic presentation. The rare combinations of less common manifestations of MEN1 are known as ‘phenocopy variants’ (2).

Germline heterozygous loss-of-function mutations of the tumor suppressor *MEN1* gene – the main molecular defect causing the MEN1 syndrome – have been detected in about 70–80% and 30% of patients with familial and sporadic MEN1 respectively (3). The percentage in familial MEN1 rises to 90% if a search for large germline deletions is performed (4, 5, 6). Thus, a definite proportion of familial and sporadic MEN1 patients do not carry *MEN1 *mutations, indicating that other tumor-susceptibility genes may be involved in the pathogenesis of this syndrome. Following the identification of a germline change in the cyclin-dependent kinase (CDK) inhibitor *Cdkn1b* gene as the causative mutation of a variant of both MEN1 and MEN2 human syndromes in a rat colony (MENX syndrome), mutations of the human homologue *CDKN1B* were searched for in individuals with the MEN1 clinical phenotype, but without *MEN1* mutations (7). Nine different germline mutations in the coding, as well as in the 5′-UTR of the *CDKN1B* gene have been described in patients with familial or sporadic MEN1-like syndromes, but negative for *MEN1* mutations (7, 8, 9, 10, 11, 12, 13). Results from *in vitro* studies have confirmed the pathogenic role of these mutations (14).

The cases with mutations of *CDKN1B* are now classified as having the MEN4 syndrome (OMIM #610755), although they do not have any peculiar phenotypic manifestations compared with MEN1-mutation-positive cases. However, given the small number of reported MEN4 patients, the clinical penetrance of the disease and the precise tumor spectrum of the syndrome are still to be defined.

The *CDKN1B *gene encodes a nuclear protein named P27 (also known as KIP1), a member of the CDK inhibitors family. P27 regulates the transition of G1 phase to S phase by inhibiting the activity of CDKs and by promoting exit from the cell cycle. The subcellular localization of P27 appears to be central to regulation of its function. The antiproliferative role of P27 depends on its presence in the nucleus, the cellular compartment where it complexes with its target kinases (cyclin E-CDK2 and cyclin A-CDK2). Conversely, in the cytoplasm, despite being deprived of its tumor suppressor role, P27 drives pro-oncogenic functions, such as apoptosis and cell motility, and promotes cell proliferation by complexing to cyclin D/CDK4,6 (15, 16). P27 harbors a CDK-binding domain in the N-terminal half of the protein, which is necessary for the mediation of the CDK-inhibitory functions. Moreover, P27 contains a bipartite nuclear localization signal (NLS) at amino acids 152/153 and 166/168 in its C-terminal part. Although *CDKN1B* is considered to be a tumor suppressor gene, somatic loss-of-function mutations in this gene have rarely been detected in different cancers (17, 18, 19). Conversely, loss or decreased expression of the P27 protein has been reported in many human cancers, where it is often associated with a poor prognosis (20). Transcriptional, translational, and post-translational modifications of P27 (i.e., phosphorylation events), leading to mis-localization and/or sequestration of the P27 protein in the cytoplasm and subsequent degradation of the protein by proteasome-dependent mechanisms, account for the loss of its physiological role in the control of the cell cycle (21, 22). Nonetheless, biallelic inactivation of *CDKN1B *is a rare event, suggesting that haploinsufficiency may explain the tumorigenic progression (23, 24, 25, 26). The *CDKN1B* gene is therefore considered to be an atypical tumor suppressor gene.

We previously reported a novel loss-of-function mutation of the *CDKN1B* gene detected in a Sardinian kindred with MEN4 (27, 28). The index case was later seen elsewhere and the detailed description of the patient has been recently published (29). Herein, we extend the genetic analysis and describe the functional characterization of the mutation.

## Materials and methods

Informed consent was obtained from the patient and one of the proband's three sons for all procedures used in this study. Our internal review board approved the study.

### Tissue samples

Formalin-fixed paraffin embedded (FFPE) parathyroid tissue (superior right gland) removed at the second parathyroid surgery was retrieved from pathological archives.

### Genetic studies

DNA was extracted from index patient's peripheral leukocytes with a Maxwell 16 Instrument according to the manufacturer's instructions (Promega Corp.). FFPE tissues were manually microdissected from two sections and samples were submitted to xylene deparaffination and then lysed and digested with proteinase K. DNA extraction was performed using the spin column procedure (QIAmp minikit; Qiagen). As no mutations in *MEN1* were identified, the genetic analysis of the coding region and intron/exon boundaries of the *CDKN1B* (NM_004064.3) gene was carried out using the BigDye Sequencing Reaction Kit v.1.1. The reaction products were separated on an ABI 3130XL automatic sequencer (Applied Biosystems). To assess for loss-of-heterozigosity (LOH), the fragment of interest in the tumoral DNA was sequenced.

### Immunohistochemistry

The immunohistochemical analysis was performed using the avidin–biotin–peroxidase complex method with the Ventana Benchmark immunostaining system (Ventana Medical System, Tucson, AZ, USA) following the manufacturer's instructions. A monoclonal anti-P27 antibody (clone SX53G, Ventana Medical System) was used to detect expression of P27. A negative control was included in the analysis by omitting the primary antibody. For the assessment of immunoreactivity, at least ten randomly selected fields under high-power magnification (×20) were chosen. In each experiment, adjacent normal parenchyma and/or infiltrating lymphocytes served as an internal positive control. Two specimens of normal parathyroid gland, inadvertently removed from normocalcemic subjects undergoing surgery for nontoxic multinodular goiter, were also used as controls. The cells were scored as positive if specific nuclear staining was detected, independently of the intensity of staining. Tumor staining was quantified according to the percentage of cells showing specific nuclear staining.

### Transfection studies

The *CDKN1B* mutation identified in the proband by sequencing was introduced by site-directed mutagenesis (Quikchange II Site-Directed Mutagenesis Kit, Stratagene, La Jolla, CA, USA) into the full-length WT human *CDKN1B* cDNA cloned in a pEYFP (EYFP, enhanced yellow fluorescent protein) plasmid backbone. The mutant protein was expressed in human cervix adenocarcinoma (HeLa) and rat pituitary epithelial-like tumor (GH3) cell lines, maintained in DMEM media supplemented with 10% fetal bovine serum, 20 mM l-glutamine, 100 units/ml of penicillin G sodium, and 100 μg/ml streptomycin, or F-12K medium supplemented with 15% horse serum, 2.5% fetal bovine serum, 20 mM l-glutamine, 100 units/ml of penicillin G sodium, and 100 μg/ml streptomycin, respectively (Invitrogen). Transient transfections were carried out as reported previously (7). For western blotting and immunofluorescence analyses, the resulting vector expressed the mutant P27 protein as a fusion protein with the YFP tag at the N-terminus. Indirect immunofluorescence was performed using a monoclonal anti-P27 antibody (BD Biosciences, San Jose, CA, USA) as reported previously (7).

## Results

### Case report

The relevant clinical details of the index case are briefly reported. Primary hyperparathyroidism (PHPT) was firstly diagnosed in the index case, a 41-year-old woman (serum calcium 11.65 mg/dl (8.4–10.2 mg/dl) and parathyroid hormone (PTH) 189 pg/ml (10–65 pg/ml)). In the same year, she underwent inferior left and right parathyroidectomy (PTx). The two superior parathyroid glands appeared normal at neck exploration and both were biopsied. The histological examination of the inferior parathyroid glands showed an oxyphil chief cells adenoma. The biopsied superior parathyroid glands were histologically normal. Serum calcium levels normalized after surgery and no further biochemical testing was performed until the age of 48 years when a relapse of PHPT was diagnosed (serum calcium 11.3 mg/dl and PTH 201 pg/ml). Two years later, at the age of 50 years, the patient was referred to the Endocrine Unit of the University Hospital of Cagliari, where she was followed from February 2007 to October 2011. The clinical and biochemical evaluation confirmed the diagnosis of relapsing PHPT and the patient underwent a second neck exploration. The superior right parathyroid gland was removed and the histological examination showed a 20 mm oxyphil chief cells adenoma. A search for mutations of the *MEN1 *gene yielded negative results. The subsequent follow-up was notable for the evidence of gastro–entero–pancreatic NETs, which were initially treated with proton-pump inhibitors and somatostatin analogs, and then successfully by surgery. At follow-up evaluation in October 2011, a relapse of PHPT was evident. Total serum calcium and PTH were 10.6 mg/dl and 138 pg/ml respectively. A 99 m-Tc-Sestamibi scan showed an uptake in the left paratracheal region. Anterior pituitary function was normal and a pituitary MRI showed slight enlargement of the left side of the gland, in the absence of focal lesions. Surveillance was advised.

In April 2012, the patient was referred to the University Hospital of Florence where in November 2012 the left superior parathyroid gland was removed and a parathyroid fragment implanted in the non-dominant forearm (29).

### Genetic analyses

The search for mutations in *MEN1* in the entire coding region and splice sites, previously performed, gave negative results. This prompted us to search for mutations of the *CDKN1B *gene and a novel germline heterozygous deletion was found in exon 1 of the *CDKN1B *gene, c.374_375delCT (according to the latest Human Genome Variation Society nomenclature –http://www.hgvs.org/mutnomen; the nucleotide numbering reflects coding DNA, with +1 corresponding to the A of the ATG translation initiation codon in the reference sequence). The two-nucleotide deletion causes a frameshift in the coding sequence, leading to a substitution of a serine (TCT) with a stop codon (TGA) and the production of a truncated P27 protein (S125X), consisting of 124 rather than 198 amino acids of the WT protein. The P27_S125X protein lacks the C-terminal domain, which contains the NLS required to enter the nucleus where the protein exerts its CDK-inhibitory function.

The proband's tumoral parathyroid tissue did not show allelic loss, because both WT and mutant alleles were demonstrated to be present by sequencing the somatic DNA. Genetic testing for the *CDKN1B* mutation was advised to all first-degree relatives, but only one of the three proband's sons agreed to be investigated. The results of the genetic test were negative.

### Immunohistochemistry

**Figure 1:**
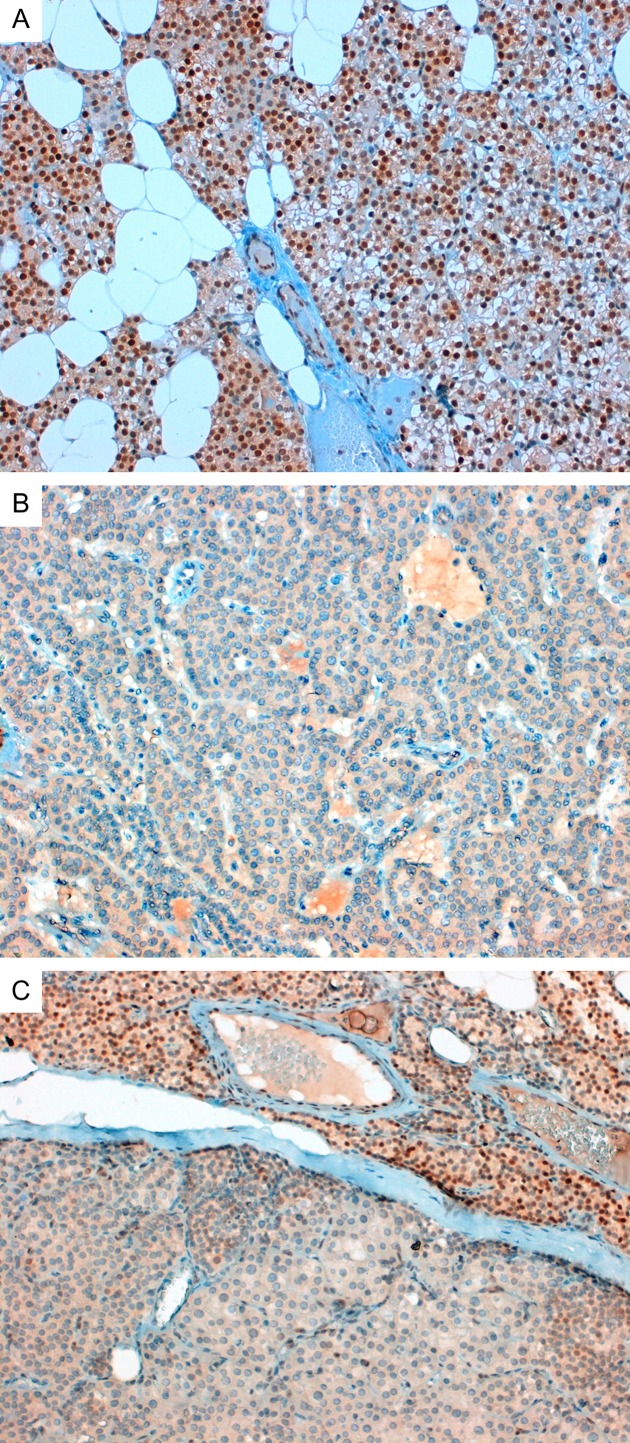
Immunohistochemical staining for P27. (A) Normal parathyroid gland from a control individual. The parathyroid cells show a diffuse nuclear immunoreactivity (×20). (B) Superior right parathyroid adenoma removed at second parathyroidectomy from the MEN4 proband scored as negative (×20). (C) Image obtained of a different area of the same section as (B) showing a rim of normal parathyroid tissue (upper part of the image) with a diffuse nuclear staining adjacent to the parathyroid adenoma (lower part) (×20).

A complete loss of nuclear P27 expression was observed in the parathyroid tumor of the proband. The adjacent normal parathyroid tissue showed strong nuclear staining (the percentage of positive cells ranging between 50% and 100%) similar to that observed in normal parathyroid tissue. Representative images are shown in Fig. 1.

### Transfection studies

**Figure 2:**
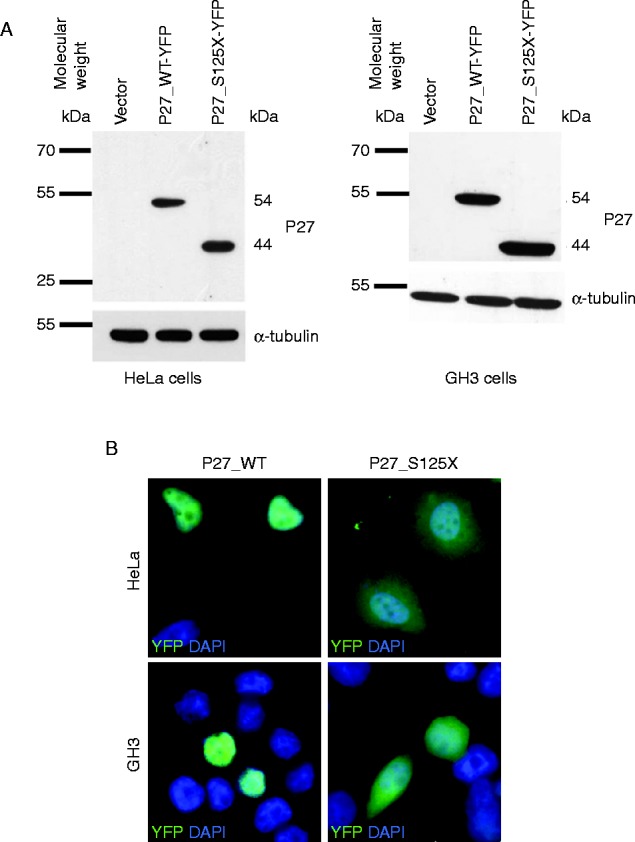
Expression and subcellular localization of exogenous proteins in HeLa and GH3 cells transfected with the indicated constructs. GH3 cells do not express endogenous P27. (A) Expression of proteins in transfected cells. 50 μg total protein obtained from cell lysates were separated by electrophoresis, blotted, and probed with YFP tagged-antibodies. Results from immunoblotting indicate that the fusion proteins are equally expressed. To control for equal loading of lysates, the membrane was probed with the anti-tubulin MAB. As expected, the P27_S125X protein, lacking 73 amino acids, is approximately 10 kDa smaller as compared with the P27_WT fusion protein. (B) Subcellular localization of YFP-tagged P27 proteins. Both HeLa (upper panels) and GH3 (lower panels) cells were fixed 24 h after transfection and nuclei were counterstained with 4′,6-diamidino-2-phenylindole (DAPI). The cells were examined for direct YFP fluorescence. P27_S125X localized mainly in the cytoplasm, whereas P27_WT was in the nucleus.

To determine the effect of mutation of human P27 on protein localization, we generated WT P27 and P27_S125X YFP-tagged proteins. The results of the western blotting analysis indicated that the fusion proteins were expressed at equal levels (Fig. 2A). As expected, the P27_S125X protein was smaller as compared with the WT P27 fusion protein. This phenomenon was reproducibly observed in HeLa and GH3 cell lines and for independent DNA clones of the same construct. We determined the cellular localization of the fusion proteins by using an anti-YFP and indirect immunofluorescence. In both cell lines, P27_WT localized in the nucleus, whereas the P27_S125X protein was retained in the cytoplasm (Fig. 2B).

## Discussion

The medical history of the patient described herein (multiglandular PHPT and multiple gastro–entero–pancreatic tumors) was consistent with the diagnosis of MEN1 syndrome, but unexpectedly, the genetic testing of the *MEN1 *gene gave a negative result. The search for mutations of other genes involved in parathyroid tumorigenesis allowed the identification of a c.374_375delCT germline mutation in the *CDKN1B *gene, leading to the diagnosis of MEN4. The age at diagnosis of PHPT in our patient (41 years), which was similar to those previously reported for other MEN4 cases carrying nonsense or frameshift mutations of *CDKN1B* (W76X and K25fs), was older than the mean age of diagnosis of PHPT in patients with MEN1 syndrome (25 years) (30), but younger than that of MEN4 patients harboring missense (56 years) and 5′UTR mutations in *CDKN1B* (67 years) (31). No other phenotypic differences were observed between patients with MEN4 carrying the c.374_375delCT truncating mutation or other mutations of *CDKN1B*.

**Figure 3:**
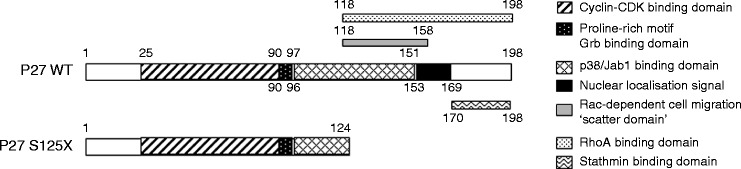
Schematic representation of WT and truncated P27_S125X protein. The binding domains to major interacting partners of P27 are represented as boxes filled with different patterns. Figures indicate the positions of the first and the last amino acid of each domain. The truncated P27_S125X protein lacks the C-terminal half of the protein, thereby loosing the nuclear localization signal, and the binding sites for RhoA, Stathmin, and Rac-dependent cell migration (also known as the ‘scatter domain’) and part of the binding site of p38Jab1.

Herein we report the functional characterization of the c.374_375delCT* CDKN1B* germline mutation. HeLa and GH3 cells transfected with the *CDKN1B* WT or mutant cDNA efficiently translated the constructs and the fusion proteins were expressed at equal levels, as demonstrated by western blotting analysis. The mutated protein was shorter (lacking the last 74 amino acids) compared with the WT protein and lacked the highly conserved C-terminal domain, which includes the bipartite NLS (amino acids 152/153 and 166/168). This protein abnormality would be predicted to result in its retention in the cytoplasm and loss of tumor-suppressive function. In addition, the lack of the C-terminal domain would also be predicted to result in the loss of the binding domains of for some cytoplasmic interacting partners of P27 involved in the regulation of cellular functions independent of progression of the cell cycle, such as differentiation and migration (Fig. 3). As a matter of fact, the results of *in vitro* studies in HeLa cell lines indicated that the P27_S125X protein was localized in the cytoplasm, confirming that the mutant protein had lost its ability to transfer into the nucleus. This *in vitro* phenotype is similar to that of cells transfected with sequence encoding the W76X nonsense variant found in the first MEN4 patient, affected by PHPT and acromegaly (7). Notably, both the S125X and the W76X mutations have previously been detected in a somatic setting in a small intestine NET (SI-NET) case and an adult T-cell leukemia/lymphoma, respectively, strengthening support for their role in tumorigenesis (17, 19).

Results from immunohistochemical studies were indicative of a complete loss of nuclear expression of P27 in the patient's parathyroid adenoma. No staining was evident in the cytoplasm. Conversely, a strong P27 staining was retained in a rim of normal parathyroid tissue surrounding the adenoma. A reduction in P27 protein in a different parathyroid adenoma obtained from the same patient at a later parathyroid surgery was also observed (29). The same authors found significant overexpression of *CDKN1B* mRNA compared with *CDKN1B*-non-mutated parathyroid tumors and normal parathyroid. The cDNA sequencing of the patient's tumoral mRNA revealed the presence of the WT, but not the mutated mRNA. No explanation is given for the lack of transcription of the mutated allele. The author's conclusion was that ‘in this case, the downregulation of the P27 protein could be at a post-transcriptional and/or post-translational level’. Only one study has previously evaluated the sequence of mRNA in a renal angiomyolipoma and normal renal tissue from a patient with MEN4 harboring the W76X mutation (7). The authors found that the WT and the W76X mutated alleles were equally transcribed in both tissues, indicating that both alleles were translated. This is in keeping with the results of our *in vitro* studies, which indicated that the P27_S125X mutant construct was fully translated into a truncated protein.

The presence of a hemizygous deletion of the *CDKN1B* gene in human hematopoietic malignancies, ovarian and prostate cancers, associated with a reduced expression of P27 is indicative of a *CDKN1B *haploinsufficient behavior in those tumors (25). Animal models also provide direct evidence of the role of P27 haploinsufficiency in the development of cancer and may explain the later onset of tumors in hemizygous compared with homozygous deficient mice (23, 32).

Haploinsufficiency of *CDKN1B* has not been clearly demonstrated in MEN4-associated tumors. Results of LOH studies, using either microsatellites flanking *CDKN1B* or sequencing the tumoral DNA, revealed allelic loss only in two out of five MEN4-associated tumors (a bronchial carcinoid and a small-cell neuroendocrine cervical carcinoma) (8, 10), but not in a parathyroid adenoma coexistent in the patient with bronchial carcinoid (10). The complete loss of P27 protein according to immunohistochemistry in these LOH-positive tumors indicates that *CDKN1B* behaves as a classical tumor suppressor gene. Conversely, the reduced expression of P27 protein in two of the three remaining LOH-negative tumors is indicative of a haploinsufficient behavior. Finally, the lack of P27 expression in the third LOH-negative case (W76X) indicates that a second somatic hit, other than LOH, inactivates the WT allele.

Both pathogenetic mechanisms (haploinsuffiency or tumor-suppressor behavior) appear to be operating in asynchronous parathyroid adenomas of the patient described herein. Indeed, our finding of a complete loss of P27 protein, in the absence of allelic loss, is indicative of an alternative somatic hit (genetic or epigenetic) at the *CDKN1B *locus. Conversely, the reduction in the level of P27 protein observed in a previous study (29) in the absence of allelic loss, is indicative of haploinsufficiency. This observation is in keeping with the finding of different somatic events in a patient with recurrent PHPT harboring a germline *CDC73* mutation (33). The positive P27 staining in the rim of normal tissue surrounding the parathyroid adenoma in our patient provides strong support for the occurrence of a second somatic hit at the *CDKN1B* locus.

In conclusion, our results reveal the pathogenic role of the c.374_375delCT* CDKN1B *germline mutation in a patient with MEN4. The absence of allelic loss and complete lack of nuclear P27 expression in the parathyroid adenoma indicate that a second somatic hit, other than LOH, may inactivate the WT allele.
